# Structural perspectives on recent breakthrough efforts toward direct drugging of RAS and acquired resistance

**DOI:** 10.3389/fonc.2024.1394702

**Published:** 2024-05-22

**Authors:** Jameela Lokhandwala, Tracess B. Smalley, Timothy H. Tran

**Affiliations:** ^1^ Department of Molecular Oncology, H. Lee Moffitt Cancer Center and Research Institute, Tampa, FL, United States; ^2^ Chemical Biology Core, H. Lee Moffitt Cancer Center and Research Institute, Tampa, FL, United States

**Keywords:** KRAS G12D, KRAS G12C, combination therapy, structural analysis, secondary mutation, acquired resistance, covalent inhibitor, non-covalent inhibitor

## Abstract

The Kirsten rat sarcoma viral oncoprotein homolog (KRAS) is currently a primary focus of oncologists and translational scientists, driven by exciting results with KRAS-targeted therapies for non-small cell lung cancer (NSCLC) patients. While KRAS mutations continue to drive high cancer diagnosis and death, researchers have developed unique strategies to target KRAS variations. Having been investigated over the past 40 years and considered “undruggable” due to the lack of pharmacological binding pockets, recent breakthroughs and accelerated FDA approval of the first covalent inhibitors targeting KRAS^G12C^, have largely sparked further drug development. Small molecule development has targeted the previously identified primary location alterations such as G12, G13, Q61, and expanded to address the emerging secondary mutations and acquired resistance. Of interest, the non-covalent KRAS^G12D^ targeting inhibitor MRTX-1133 has shown promising results in humanized pancreatic cancer mouse models and is seemingly making its way from bench to bedside. While this manuscript was under review a novel class of first covalent inhibitors specific for G12D was published, These so-called malolactones can crosslink both GDP and GTP bound forms of G12D. Inhibition of the latter state suppressed downstream signaling and cancer cell proliferation *in vitro* and in mouse xenografts. Moreover, a non-covalent pan-KRAS inhibitor, BI-2865, reduced tumor proliferation in cell lines and mouse models. Finally, the next generation of KRAS mutant-specific and pan-RAS tri-complex inhibitors have revolutionized RAS drug discovery. This review will give a structural biology perspective on the current generation of KRAS inhibitors through the lens of emerging secondary mutations and acquired resistance.

## Introduction

The Kirsten rat sarcoma viral oncogene homolog (KRAS) is the most frequently mutated oncogene in cancers, and mutations in the RAS family affect approximately 30% of cancer diagnoses (1, 2). KRAS, HRAS and NRAS belong to a superfamily of small GTP-binding proteins known as GTPases, which control key biological processes such as cell division, cell differentiation, and apoptosis (3, 4). The regulation of RAS proteins is cyclic, alternating between on-GTP and off-GDP bound states, acting as a molecular switch to downstream signaling pathways (5). Common oncogenic mutations in G12, G13, and Q61 disrupt GTP hydrolysis (6, 7), ultimately locking KRAS in an active GTP-bound state that leads to the continuous activation of downstream signaling and promotion of tumor proliferation (8). Consistent within the RAS protein family, RAS activation and inactivation are controlled by RAS-guanine nucleotide exchange factors (RAS-GEFs) and RAS-GTPase activating proteins (RAS-GAPs), respectively, which in turn are controlled by receptor tyrosine kinases (RTKs) and cytokine receptors (9–12). The GTP-loaded RAS can interact with the RAS-binding domains (RBDs) in downstream effector proteins such as members of Rapidly Accelerated Fibrosarcoma (RAF) kinases ARAF, BRAF, and CRAF to activate further signaling (13). Understanding the central role that RAS plays in cancer signaling pathways has led to the creation of the NCI RAS Initiative nearly a decade ago, spearheaded by Dr. Frank McCormick and Dr. Dwight Nissley (14). This initiative is solely dedicated to the investigation of RAS cancer biology and the development of the RAS drug discovery program, highlighting the paramount importance of RAS as a direct drug target.

## Discovery of KRAS^G12C^ inhibitors paving the way for drugging the undruggable

**Figure 1:**
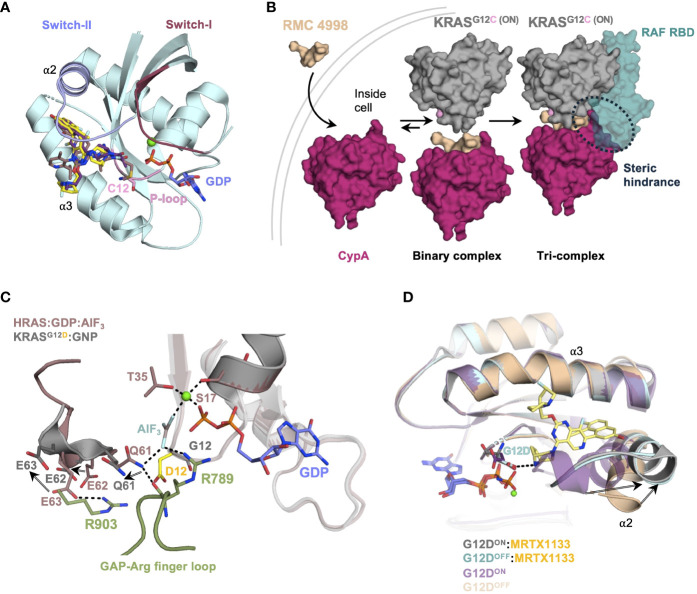
**(A)** Crystal structure (PDB: 6OIM, 7RPZ, and 6UT0) of KRAS^G12C/D^ in complex with Adagrasib (purple) and Sotorasib (salmon) and MRTX-1133 (yellow). The switch-I and switch-II regions are highlighted in pink and blue. **(B)** Schematic of tri-complex formation by RMC-4998 a G12C^ON^ inhibitor, the tri-complex crystal structure of KRAS^G12C^:CypA : RMC-4998 (PDB: 8G9P) was aligned to KRAS : CRAF^RBD/CRD^ complex structure (PDB: 6XI7). **(C)** Crystal structure of the complex between H-RAS and GTPase activating protein (GAP-334, green) in the active transition state (PDB: 1WQ1, gray) was aligned to the crystal structure of KRAS^G12D^ in its active state (PDB: 5USJ, cyan). **(D)** G12D^OFF^ (PDB: 7RPZ, pale cyan) and G12D^ON^ (PDB: 7T47, gray) crystal structures in complex with MRTX-1133 are aligned to the G12D^ON^ (PDB: 5USJ, purple) and G12D^OFF^ (PDB: 5US4, wheat) structures.

Interestingly, KRAS was first discovered in the early 1980s, yet there were no approved targeted drugs until 2021. In 2013, the Shokat laboratory achieved a breakthrough in this field using fragment screening and discovered small molecules capable of covalently binding to KRAS^G12C^ mutant at a previously unidentified allosteric switch-II pocket (15). Following this discovery, the first generation of small molecule inhibitors to KRAS^G12C^ from Amgen (Sotorasib) and Mirati (Adagrasib) therapeutics, were developed to covalently target the reactive C12 of KRAS^G12C^ (16, 17). Both inhibitors have shown some efficiency in metastatic sites. Recent reports discussing the CodeBreaK (Sotorasib) and Krystal (Adagrasib) clinical trials have illustrated effectiveness in central nervous system metastatic tumors, making them attractive candidates for later stage treatment (18). Sotorasib and Adagrasib bind to GDP-bound KRAS (inactive) and occupy the allosteric switch-II pocket located between the central β sheet, α2 (switch-II) and α3 helices of KRAS (**Figure 1A**, **Table 1**). Both compounds disrupt SOS-mediated nucleotide exchange of GDP for GTP (19, 29–31). The development of this lock and key inhibition of KRAS^G12C^ has laid the foundation for the development of KRAS-targeted therapies and has generated opportunities for future drug campaigns focusing on other mutant forms of KRAS.

**Table 1:** Summary of RAS inhibitors in this review.

Drug	Target	Kd	Mechanism	Pocket	State	Citation
Sotorasib (AMG510)	G12C	–	Covalent	Switch-II	Inactive	(19)
Adagrasib (MRTX849)	G12C	–	Covalent	Switch-II	Inactive	(16)
MRTX-1133	G12D	(0.2pM)^OFF^	Non-covalent	Switch-II	Active/Inactive	(20)
RM-018	G12C, Y96D	NA	Covalent and steric blockade	Switch I and II	Active	(21)
RMC-6236	Pan-RAS	NA	Non-Covalent Steric blockade	Switch I and II	Active	(22)
RMC-6291	G12C	NA	Covalent and steric blockade	Switch I and II	Active	(23)
RMC-7977	Pan-RAS	CypA (195nM) KRAS^WT^ (120nM) KRAS^G12V/C/D/R^ (40-270nM) NRAS^WT^ (98nM) HRAS^WT^ (90nM)	Non-covalent and steric blockade	Switch-I and II	Active	(24)
RMC-9805	G12D	NA	Covalent and blockade	Switch I and II	Active	(25)
BI-2852	*Pan-RAS	KRAS^WT^ (7.5µM)^ON^/(1.1µM)^OFF^ KRAS^G12D^ (740nM)^ON^/(2.0µM)^OFF^ HRAS^WT^ (570nM)^ON^/(2.5 µM)^OFF^ NRAS^WT^ (1.37µM)^ON^/(8.3µM)^OFF^	Non-covalent	Between switch I and II	Active and inactive	(26)
BI-2865	Pan-KRAS	KRAS^WT^ (6.9nM) KRAS^G12C^ (4.5nM) KRAS^G12D^ (32nM) KRAS^G12V^ (26nM) KRAS^G13D^ (4.3nM)	Non-covalent	Switch-II	Inactive	(27)
Malolactone G12Di	G12D	–	Covalent	Switch-II	Active and inactive	(28)

*Presumably.

Active/Inactive (ON/OFF).NA, Not applicable.

## Targeting KRAS^G12C^ active state with molecular glue inhibitors

The early inhibitors such as Adagrasib and Sotorasib, only target the inactive GDP-bound form of KRAS, leaving a need to develop active KRAS inhibitors. Targeting the active GTP-bound KRAS remained difficult until four years ago, when Revolution Medicines came out with tri-complex covalent G12C^ON^ inhibitor, RM-018 (32, 33). This inhibitor is a unique molecular glue, which forms a trimeric complex with Cyclophilin A (CypA) and KRAS^G12C(ON)^. RM-018 is highly selective for the active form of KRAS^G12C(ON)^ with the bispecific utility of a RAS ligand and CypA ligand. The RAS ligand specific for G12C exploits a non-covalent interaction along with a warhead that forms a covalent linkage with G12C side chain as seen by several G12C inhibitors. CypA ligands consist of molecules derived from natural products, such as Sanglifehrin A, that bind strongly and reversibly to a ubiquitously expressed chaperone protein CypA (**Figure 1B**) (34). The innovative design of RM-018 entails remodeling the surface of CypA and has a high affinity and selectivity for the active state of KRAS^G12C^ (23). Upon cell entry, RM-018 initially forms a complex with cyclophilin A (CypA) (**Figure 1B**, **Table 1**). This binary complex then exploits non-covalent interactions with the switch-I and switch-II region of KRAS and facilitates a facile reaction with the G12C side chain to form a stable trimeric complex, which prevents effectors and regulators from binding to G12C^ON^ (21, 32, 33).

## Targeting KRAS^G12D^ mutant with non-covalent and covalent inhibitors

Due to its high prevalence in various cancers- 40% in colorectal, 17% in lung adenocarcinomas, and a staggering 51% in pancreatic ductal adenocarcinoma (PDAC)- the G12D mutant has emerged as a major target of interest (35–37). This KRAS variant exhibits a low rate of both intrinsic (~2.5-fold lower than G12C variant) and GAP-mediated GTP-hydrolysis, further burdening the cell with hyperactive mutant KRAS (38, 39). The side chain of G12D, in any given rotameric conformation, prevents the key arginine finger of GAP from inserting into the active site of RAS to participate in the transition-state coordination of the GAP-mediated GTP hydrolysis reaction (6, 40–42). Additionally, the negative point charge of the G12D side chain constantly exerts a repulsive force radially outward, especially toward the negatively charged E62 and E63 of the catalytic loop. Together, E62 and E63 with the key catalytic residue Q61, are pushed away from their catalytically competent position for GTP hydrolysis (**Figure 1C**). These are the structural and physical bases for substantially impaired GAP-mediated and intrinsic GTP hydrolysis in KRAS^G12D^, accounting for the highest rate of lethality among RAS-driven cancers.

The negatively charged G12D mutation has been considerably challenging to target compared to other G12 mutations. Drugs designed to specifically target G12D must be able to form a covalent bond or a hydrogen bond or an electrostatic interaction with the carboxylate group of G12D side chain, although the latter is unlikely to be a good candidate due to the plasma membrane barrier. As for the warhead design, G12D side chain, a weak nucleophile, is not as reactive compared to a cysteine residue. Hence, a drug design specific for aspartate requires a novel class of electrophiles that can execute a facile, one-step coupling reaction, similar to that of click chemistry or cysteine oxidation/S-C bond formation, with the G12D side chain. In addition, there are many other aspartate and glutamate residues on the protein surface of RAS and all other cytosolic proteins in the cell, which makes it very challenging to selectively target KRAS^G12D^. Therefore, drug discovery targeting KRAS^G12D^ requires a novel chemistry, a highly specific pocket design, and possibly point mutations of other solvent-exposed aspartate/glutamate residues. As mentioned above, the Shokat laboratory just released the first structure of G12D in which the carboxylate of G12D side chain is alkylated with a lactone-based electrophile or malolactone (**Table 1**) (28). The novelty of this compound design takes advantage of the 4-membered ring strain of heterocyclic lactone in a pseudo-staggered conformation. This exposes the lowest unoccupied molecular orbital of the C-O bond and facilitates a direct strain-release S_N_2 ring-opening attack by G12D side chain. In addition, the authors’ judicious choice of substituents on the α-carbon of the enantioselective malolactone shielded the compound from ~55 M of competing water molecules present in the buffer. For more details, readers can examine the reaction trajectory from the reference therein (28).

Fortuitously, the G12C cancer variant has provided us with a foundation to target G12D using G12C pharmacophores. The authors of the MRTX-1133 have successfully synthesized a compound that forms a hydrogen bond with the G12D side chain via a nitrogen donor (20). This crucial hydrogen bond is critical for the specificity and anchoring of the drug, allowing the rest of the ligand to “wiggle” and adjust, taking advantage of the dynamic nature of the switch-II for induced fit. In the absence of direct ligand:G12D side chain interaction, the specificity would be significantly reduced and require additional stabilization. Since the overall design of MRTX-1133 was based on Adagrasib, other cancer drugs targeting G12C can also be modified to be reactive against G12D-driven cancers.

Interestingly, MRTX-1133 has been shown to inhibit both inactive and active forms of G12D (43). More surprisingly, superposition of both G12D^ON^ and G12D^OFF^ structures in complex with MRTX-1133 revealed that MRTX-1133 has the same binding mode, even though GDP and GTP-bound structures have distinct conformations in the absence of MRTX-1133 (**Figure 1D**). This suggests that MRTX-1133 induces a novel binding mechanism. Incidentally, the critical hydrogen bond between MRTX-1133 and G12D side chain also alleviates the repulsive force of G12D point charge toward the catalytic loop as mentioned above (**Figure 1C**). Therefore, in addition to hindering G12D interaction with GAPs, MRTX-1133 or any drug compound that interacts with G12D^ON^ via a hydrogen bond or a covalent bond could enhance or restore its intrinsic GTP hydrolysis, allowing G12D^ON^ to return to its inactive GDP-bound form and reducing G12D^ON^ concentration in the cell.

With the advent of the novel class of G12D-specific covalent inhibitors, malolactones, a combinatorial arsenal of compounds can be designed to combat G12D-driven tumors, as the original authors have already demonstrated in cellular context as well as in mouse xenografts (**Table 1**). As is the case with G12C^ON^, covalent KRAS^G12D^ specific inhibitors can be made by the tri-complex platform. This is perhaps the most remarkable breakthrough given the challenges of targeting G12D highlighted earlier. During a recent AACR conference, Revolution Medicines announced its sister version of G12C^ON^ tri-complex inhibitor, RMC-9805, targeting G12D^ON^ (**Table 1**) (25, 44, 45). While the structure for this version has not been published, its overall structure is most likely similar to other tri-complex inhibitors, with the exception that the G12D warhead is expected to carry a novel aspartate-reactive electrophile similar to malolactones. This would make RMC-9805, together with malolactones, the first class of covalent inhibitors specific for the carboxylate group of the G12D side chain and a very promising alternative to MRTX-1133 targeting KRAS^G12D^ driven cancers.

## Pan KRAS/RAS inhibitors- targeting all Ras isoforms and mutants with one stone

**Figure 2:**
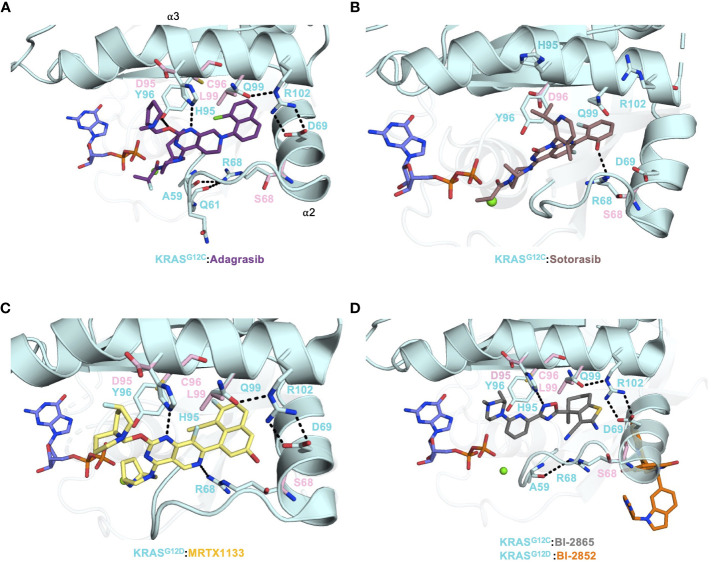
**(A)** Crystal structure of KRAS^G12C^ (PDB: 6UT0) in complex with Adagrasib (purple) and the secondary mutations were modeled on the structure and shown as pink sticks. **(B)** Crystal structure of KRAS^G12C^ (PDB: 6OIM) in complex with Sotorasib (salmon) and the secondary mutations were modeled on the structure and shown as pink sticks. **(C)** Crystal structure of KRAS^G12D^ (PDB: 7RPZ) in complex with MRTX-1133 (yellow) and the secondary mutations were modeled on the structure and shown as pink sticks. **(D)** The crystal structure of KRAS^G12C^ (PDB: 8AZR) in complex with pan-KRAS inhibitor BI-2865 (gray) and crystal structure KRAS^G12C^ (PDB: 6GJ8) in complex with BI-2852 (orange) were aligned and the secondary mutations were modeled on the structure and shown as pink sticks.

Boehringer Ingelheim recently published a pan-KRAS-selective inhibitor, BI-2865, which was designed based on G12C pharmacophores targeting the inactive form of KRAS and accompanied by atomic-resolution crystal structures (27). The authors showed that BI-2865 is approximately 2-3 orders of magnitude more selective for KRAS than HRAS and NRAS, and inhibited many common G12, G13, and Q61H mutations, with the exceptions of G12R and other Q61 mutations. Based on the structure of the complex and mutagenesis studies, BI-2865 confers its selectivity around H95 (**Figure 2D**, **Table 1**), which is one of a few amino acids of the RAS G-domain that differs among RAS isoforms. However, as a pan-KRAS inhibitor, BI-2865 also inactivates WT KRAS. Presently, no clinical data demonstrating the consequences of blocking WT KRAS have been reported (46). Thus, it would be of great interest to see how cancer patients can tolerate potential toxicity of this pan-KRAS inhibitor. Nevertheless, WT KRAS activity can be potentially compensated by NRAS and HRAS isoforms.

Several other pan-RAS inhibitors have also been reported (47–49). However, none of them have co-crystal structures. A few years earlier, Boehringer Ingelheim unveiled its first, presumably*, pan-RAS compound BI-2852 that bound and inhibited KRAS at a site distinct from the switch-II pocket, as shown in a series of KRAS^G12D^ structures (**Figure 2D**, **Table 1**) (26). BI-2852 was shown to bind stronger to KRAS^G12D^ than WT KRAS, but it also bound to NRAS and HRAS almost as tight as KRAS^G12D^ and inhibited WT KRAS, NRAS, and HRAS (**Table 1**). The mechanism of inhibition of BI-2852 involves dimerization of KRAS, which prevents RAF binding (50).

In addition to KRAS mutant-specific inhibitors, Revolution Medicines also reported a tri-complex pan-RAS inhibitor, RMC-6236 or RAS^MULTI(ON)^ (**Table 1**) (22, 51, 52). To be a pan-RAS inhibitor, the RAS ligand must interact with RAS noncovalently as seen in a series of tri-complex structures of RMC-7977 (**Table 1**) (24) with different WT RAS isoforms and mutants. Additionally, emerging studies show that these broad-spectrum inhibitors may have moderate tumor selectivity in NSCLC and PDAC mouse models and decreased resistance escape (53, 54). As a versatile inhibitor against the active form of RAS, RAS^MULTI(ON)^ quickly made its way into phase I clinical trials. However, despite this unprecedented breakthrough and promising results in pre-clinical studies, data from clinical trials are needed to assess its efficacy and toxicity. Nevertheless, as expected for a pan-RAS inhibitor, RAS^MULTI(ON)^ also inhibits WT HRAS and NRAS. Thus, while RAS^MULTI(ON)^ can be practically used to treat the maximum number of patients with any RAS-driven cancer, the inhibition of all WT RAS proteins may lead to higher toxicity since there are no known compensatory mechanisms. Fortunately, even if RAS^MULTI(ON)^ exhibited undesirable clinical outcomes, tri-complex platform for specific RAS mutants mentioned above for G12C^ON^ and G12D^ON^ can be used to treat KRAS mutant-specific cancers. In addition, it is also possible to design warheads for the other oncogenic variants, G13C and Q61H, which are currently in the pipeline at Revolution Medicines. Nevertheless, it is important to note that clinical results only reflect short-term outcomes. Long-term studies are needed for these drugs to assess their effectiveness against resistance by cancer cells.

## Secondary mutations and acquired resistance

Adagrasib and Sotorasib have had unprecedented clinical success in NSCLC patients with KRAS^G12C^ mutation (~14% frequency). However, despite these advances in treatment options for patients in this subgroup, acquired resistance mechanisms have been reported (8, 13). There are several routes that cancer cells take to evade KRAS inhibition. Two avenues of note are secondary mutations occurring within the drug-binding pocket and KRAS gene amplification. In a study by Awad et al., 38 patients (27 non-small-cell lung cancer, 10 colorectal cancer, and 1 appendiceal cancer) treated with Adagrasib were investigated for acquired KRAS alterations. Out of 38 patients, 17 developed various resistance mechanisms to Adagrasib. The KRAS alterations included G12D/R/V/W, G13D, Q61H, R68S, H95D/Q/R, Y96C and the amplification of KRAS^G12C^ allele were observed in 7 patients (55). Covalent inhibitors, such as Adagrasib and Sotorasib, provide a strong anchor for drug design and development at the reactive cysteine of G12C. Hence, the observed secondary mutations from G12C to G12D/R/V/W would be most detrimental since that crucial anchor is severed.

Residues R68, H95, Y96 and Q99 of the switch-II pocket interact with G12C inhibitors and stabilize drug:protein interactions (**Figures 2A, B**). R68 side chain forms Van der Waals interactions to the piperidine and naphthyl moieties of Adagrasib (**Figure 2A**). Furthermore, R68 may also play a role in forming the switch-II pocket by holding A59 and Q61, via hydrogen bonds, in a favorable conformation for drug binding (55). R68S mutation would remove the favorable contact with Adagrasib and could disrupt binding. Both Adagrasib and Sotorasib bind to the H95 groove, in which H95 forms a critical hydrogen bond with the pyrimidine moiety of Adagrasib (**Figure 2A**) (16, 19). However, H95 does not make the equivalent hydrogen bond with Sotorasib (**Figure 2B**). Although H95D mutation could retain this hydrogen bond, its negative charge destabilizes the overall hydrophobic interactions between Adagrasib and KRAS (**Figure 2A**). In cellular studies, mutations at the H95 site were sensitive to Sotorasib whereas R68S was shown to cause resistance to Sotorasib (55). This can be explained by the fact that Sotorasib makes a critical hydrogen bond with R68 but not H95 (**Figure 2B**). Furthermore, resistant clones that arose after *in-vitro* treatment with Adagrasib and Sotorasib on Ba/F3 cells included mutations such as Q99L (56). Q99L mutation was shown to be resistant to Adagrasib but not to Sotorasib. Q99 residue is part of the α3 helix. Substituting a polar glutamine with a hydrophobic leucine resulted in a loss of a hydrogen bond that anchored the R102-D69 salt bridge connecting α2 and α3 helices, which helped confine the Adagrasib binding pocket (**Figure 2A**). However, this binding mode does not exist in Sotorasib and thus Q99L mutation did not affect Sotorasib (**Figure 2B**).

Interestingly, mutations found at the Y96 residue (to C/D/S) were reported to cause resistance to both Adagrasib and Sotorasib via *in vitro* experiments and in patients (55, 56). The structures of KRAS^G12C^ in complex with Adagrasib and Sotorasib indicate that although the hydroxyl group of Y96 interacts with water molecules at this solvent-accessible interface, its benzene ring forms extensive hydrophobic interactions with both compounds. Hence, mutations at Y96 to a smaller or charged residue (C/D/S) would sever the hydrophobic interactions. Moreover, molecular dynamic simulation studies have shown that Y96D mutation increases the flexibility of the switch-II and α3 helix region, causing it to become more dynamic and unstable, and subsequently weakening the Sotorasib binding pocket (57). Thus, the dynamic nature of the switch-II region also allows the drug to adjust and remain in sync with it. Gradually, this causes the drug to become less effective in altering the switch-II dynamics and allows RAS effectors/regulators to resume their normal RAS activities. Overall, these secondary mutations undoubtedly impart selective pressure on ligand stability and could result in breakage of the warhead in G12C (Adagrasib/Sotorasib), or hydrogen bonding interaction in G12D (MRTX-1133) or even direct G12 mutation as a mechanism of resistance. Therefore, secondary mutations affecting G12C inhibitors and specific to the switch-II pocket could arise during G12D-specific MRTX-1133 or pan-KRAS BI-2865 treatment. The four residues that harbored secondary mutations, R68, H95, Y96 and Q99, make crucial contacts with MRTX-1133 and BI-2865 and were shown to be mutated after Adagrasib or Sotorasib treatment. In fact, Adagrasib, MRTX-1133 and BI-2865, but not Sotorasib, have a very similar KRAS binding mode in the switch-II pocket (**Figures 2A, C, D**). In any event, as is the case with many cancer drugs, acquired resistance via point mutations makes KRAS a continuously moving target and must be taken into consideration in drug development.

Nonetheless, the recent breakthrough work by Revolution Medicines has demonstrated that secondary mutations can be overcome by the tri-complex platform with the parent compound of RMC-6291, RM-018, for secondary mutation KRAS^G12C/Y96D^ (21). One plausible explanation is that CypA of tri-complex inhibitors encloses the ligand and provides an extensive stabilizing interface with KRAS^G12C^, in addition to imposing an 18 kDa physical barrier to RAS effector/regulator binding. Thus, a secondary point mutation on KRAS^G12C^ is unlikely to significantly impact the vast interaction between KRAS^G12C^ and CypA. It is not surprising that RMC-6291 is a promising and potent treatment with a pre-clinical overall response rate of 72% and a disease control rate of 92% in models with KRAS^G12C^ NSCLC. Furthermore, RMC-6291 exhibits the potential for reduced resistance during treatment and is currently recruiting for Phase 1/1b clinical trials (NCT05462717) (58, 59). Notably, that while many drug developers desire a monotherapy option for patients suffering from KRAS-driven tumors, acquired resistance continues to present difficulties in treatments. These secondary mutations could continue with newly developed inhibitors that target the switch I/II pocket. In addition to KRAS secondary mutations, resistance mechanisms can be mutations in any member of the RAS signaling pathway such as upstream tyrosine receptor kinases and downstream effectors BRAF/CRAF (60). Therefore, combination therapies offer an advantage to potentially circumvent these resistance mechanisms.

## Combining immunotherapy with inhibitor treatments

The evolution of the KRAS field has yielded diverse therapeutic approaches, potentially considered for monotherapies. However, despite promising results *in vitro*, *in vivo*, and clinical studies, other strategies are needed to address the issue of acquired resistance. As KRAS has been shown to modulate the immune system, KRAS mutations have been noted to cause an immunosuppressive tumor microenvironment (TME), allowing evasion of immune detection and continued unmitigated proliferation (61–63). Several groups have expanded on novel approaches to combine immunotherapies with the current targeted approaches. These efforts are largely represented by antibody drugs, whether through bispecific T-cell engagers (BiTEs) targeting neoantigens on the cell surface or through targeting the PD-1 immunosuppressive signaling pathway (64, 65). Among these advancements, Zhang et al. leveraged the covalent bond between G12C inhibitors (ARS1620) and KRAS^G12C^ mutants to subsequently display MHC class I complexes presenting the drug-modified neoantigen on the cell surface (66). Using phage display, an antibody-fragment (P1A4) specific to this new antigen was discovered and developed into a BiTE using a CD3 T-cell targeting single-chain fragment variable (scFv – L2K-07). Zhang et al., determined that the BiTE could invoke a cytotoxic T-cell response from peripheral mononuclear blood cells in co-cultures with KRAS^G12C^ cell lines (H358, Miapaca and SW1573), which included those cells resistant to direct KRAS^G12C^ inhibition. While these studies are still in the preliminary stages of development, there is an ongoing emphasis on the importance of understanding what role different immune landscapes play in the tumor’s response to immunotherapies (67). Highlighted by this is Amgen’s early-phase clinical trial combining Sotorasib with anti-PD-1, which was inhibited by high-grade liver toxicities in many patients (68). While the cause of the toxicity is not fully understood, results still suggest that many variables need to be considered when designing combination immunotherapies. Nevertheless, novel mechanisms are emerging from preclinical research, suggesting alternative candidates to target in addition to KRAS. An example of this would be the inositol-requiring enzyme 1α branch of the unfolded protein response (IRE1α), which gets inactivated by oncogenic KRAS (69). The stabilization of IRE1α disrupts the normal programming of proteostasis mechanisms, highlighting a new druggable target. Continued efforts to synergistically combine therapeutics to KRAS inhibitors are ongoing.

## Conclusions

Exciting advances in RAS cancer biology have launched new drug campaigns aimed at treating patients with KRAS mutations. By exploiting the nucleophilicity of the G12C residue in the switch-II pocket, drug expansion efforts have led to the first FDA-approved targeted therapies for KRAS mutants. However, targeting KRAS has historically posed challenges as other mutant forms of KRAS are not susceptible to G12C-specific inhibitors. Expanding beyond the inhibition of G12C is highlighted by the success of the non-covalent inhibitor to KRAS^G12D^ having shown promising results in PDAC mouse models, and covalent G12D-specific inhibitors in mouse xenografts. Furthermore, the current perspectives in the KRAS field are positive, underscored by the next generation of small molecule tools aimed at addressing KRAS in cancer. Tools such as the active form pan-RAS and KRAS mutant-specific tri-complex inhibitors have shown promising results *in vivo* and are moving toward the clinic. With this toolbox in hand, clinicians, researchers and drug developers can address KRAS secondary mutations that are already emerging in clinical settings. Further combination therapies specifically with immunotherapy, have shown mixed but overall promising results and should continue to be pursued to determine clinical effectiveness.

## Author contributions

JL: Writing – original draft, Writing – review & editing. TS: Writing – original draft, Writing – review & editing. TT: Writing – original draft, Writing – review & editing.
